# Reduction in dynamin-2 is implicated in ischaemic cardiac arrhythmias

**DOI:** 10.1111/jcmm.12335

**Published:** 2014-08-05

**Authors:** Dan Shi, Duanyang Xie, Hong Zhang, Hong Zhao, Jian Huang, Changming Li, Yi Liu, Fei Lv, Erlinda The, Yuan Liu, Tianyou Yuan, Shiyi Wang, Jinjin Chen, Lei Pan, Zuoren Yu, Dandan Liang, Weidong Zhu, Yuzhen Zhang, Li Li, Luying Peng, Jun Li, Yi-Han Chen

**Affiliations:** a Key Laboratory of Basic Research in Cardiology of the Ministry of Education of China, Tongji UniversityShanghai, China; b Institute of Medical Genetics, Tongji UniversityShanghai, China; c Department of Cardiology, East Hospital, Tongji UniversityShanghai, China; d Department of Pediatrics, Tongji Hospital, Tongji UniversityShanghai, China; e Department of Pathology and Pathophysiology, Tongji University School of MedicineShanghai, China

**Keywords:** ischaemic cardiac arrhythmias, dynamin-2, ion channels, Nav1.5, Kir2.1

## Abstract

Ischaemic cardiac arrhythmias cause a large proportion of sudden cardiac deaths worldwide. The ischaemic arrhythmogenesis is primarily because of the dysfunction and adverse remodelling of sarcolemma ion channels. However, the potential regulators of sarcolemma ion channel turnover and function in ischaemic cardiac arrhythmias remains unknown. Our previous studies indicate that dynamin-2 (DNM2), a cardiac membrane-remodelling GTPase, modulates ion channels membrane trafficking in the cardiomyocytes. Here, we have found that DNM2 plays an important role in acute ischaemic arrhythmias. In rat ventricular tissues and primary cardiomyocytes subjected to acute ischaemic stress, the DNM2 protein and transcription levels were markedly down-regulated. This DNM2 reduction was coupled with severe ventricular arrhythmias. Moreover, we identified that the down-regulation of DNM2 within cardiomyocytes increases the action potential amplitude and prolongs the re-polarization duration by depressing the retrograde trafficking of Nav1.5 and Kir2.1 channels. These effects are likely to account for the DNM2 defect-induced arrhythmogenic potentials. These results suggest that DNM2, with its multi-ion channel targeting properties, could be a promising target for novel antiarrhythmic therapies.

## Introduction

Acute ischaemic arrhythmias are frequently encountered in clinical practice, accounting for 80% of sudden cardiac deaths 1,2. The initiation and maintenance of ischaemic arrhythmias are mainly ascribed to electrical disorders in the heart 3,4, but the mechanisms for establishing the electrical instability have not been elucidated. Disorders in membrane ion channels cause the majority of malignant arrhythmias 5–7. Gene expression, including transcription, RNA splicing and processing and post-translational modification, is an early primary step for generating functional cardiac ion channels 7. In the case of ischaemia, more significant changes may occur in the remodelling of these sarcolemma ion channels 8,9. Some of the mechanisms that govern the dynamic lifecycle of these channels, including their targeting to organized subdomains on the cardiomyocyte surface, have been identified 10; however, the molecular details for regulating sarcolemma ion channel trafficking under pathophysiological conditions remain to be investigated.

Dynamin-2 (DNM2), a GTPase implicated in membrane remodelling, has a critical role in endocytic membrane fission events 11. Our previous studies have shown that DNM2 can regulate the function and trafficking of cardiac ion channels 12,13, such as for the L-type calcium channel, indicating that DNM2 has an important role in regulating cardiac electrophysiology. We proposed that DNM2 defects contribute to ischaemic arrhythmias. In the present study, we analysed the changes in DNM2 expression in infarcted hearts and ischaemic cardiomyocytes, the association between DNM2 defects and ischaemic arrhythmias and the mechanisms underlying arrhythmogenic potentials.

## Materials and methods

### Animal model

All animal studies conform to the Guide for the Care and Use of Laboratory Animals published by the U.S. National Institutes of Health (NIH Publication no. 85-23, revised 1996). Male Sprague–Dawley (SD) rats (weighing 220–260 g) were housed in a 12 hrs dark/light cycle. Before the surgery, we anesthetized the rats with sodium pentobarbital (25 mg/kg) *via* a single intraperitoneal injection; then, we treated the rats with artificial, controlled, mechanical ventilation by trachea intubation. A left thoracotomy was performed to provide access to the heart, and a ligation was placed under the left coronary artery between the pulmonary artery out-flow tract and the left atrium. All of the sham control operations were the same, except the knots tied along on the ventricular anterior wall were not tight 14,15.

### ECG recording

A standard lead II ECG was recorded, 4 hrs after the coronary artery ligation was performed, on a data acquisition PowerLab ECG recording system (AD Instruments, Lexington, NSW, Australia). The incidence of arrhythmias was evaluated in accordance with the criteria of arrhythmias 16, with the following values: 0 = no arrhythmia; 1 = <10 sec. pre-mature ventricular contraction (PVC) and/or ventricular tachycardia (VT); 2 = 11–30 sec. PVC and/or VT; 3 = 31–90 sec. PVC and/or VT; 4 = 91–180 sec. PVC and/or VT or reversible ventricular fibrillation (VF) for <10 sec.; 5 = >180 sec. PVC and/or VT or >10 sec. reversible VF; and 6 = irreversible VF.

### Isolation of NRVMs and recombinant adenovirus transfection

Neonatal rat ventricular myocytes (NRVMs) were isolated from 2-day-old SD rats by serial trypsinization 17; the myocytes were then cultured and infected with adenovirus as previously described 12. The cDNAs for rat DNM2WT and DNM2K44A were obtained from ATCC (www.atcc.org; MBA-94: DNM2WT; MBA-95: DNM2K44A). Adenoviruses containing NC, DNM2WT and DNM2K44A were generated by Shanghai R&S Biotechnology Co., Ltd, Shanghai, China.

### Langendorff-perfused heart

Sprague–Dawley rats (220–250 g) were injected with heparin (1000 IU/kg i.p.) 20–30 min. before anesthetization with pentobarbital sodium (500 mg/kg i.p.). When a rat was successfully anesthetized, its chest cavity was opened and the heart was carefully excised and immersed in cold (4°C) Krebs–Henseleit buffer (in mM; NaCl 118.5, NaHCO_3_ 25.0, KCl 4.7, MgSO_4_ 1.2, glucose 11 and CaCl_2_ 2.5). The aortic root of isolated heart was rapidly placed onto a Langendorff system (constant pressure of 50 cm H_2_O), and the heart was washed with cold Krebs–Henseleit buffer (4°C). Then, two ECG probes were fixed on the left ventricle and auricular dextra, and a balloon was placed in the left ventricle. After 15 min. of perfusion with warm Krebs–Henseleit solution (37°C), when the heart's ECG and LV pressure remained stable, dynasore (Sigma-Aldrich, St. Louis, MO, USA) solution (15 μM) 18 was administered for 15 min.

### Real-time PCR

mRNA was extracted from the rat heart tissue using TRIzol reagent (Invitrogen, Carlsbad, CA, USA). The reverse transcription reaction was carried out with Prime script RT reagent (Takara, Seta, Otsu, Shiga, Japan), and the real-time PCR was performed in triplicate using the SYBR Green PCR Master Mix (Applied Biosystems, Warrington, UK). The primer sequences were as follows: RAT-DNM2-RT-F: 5′-CACAGCCCCACTCCACAGCG-3′ and RAT-DNM2-RT-R: 5′-GGTCCAGGCCGGGATGGGAT-3′.

### Electrophysiology

A whole-cell patch clamp was applied for ion channel current and AP recording as previously described 19. The ion channel currents were recorded with a tight-seal patch clamp in the voltage clamp mode, and APs were recorded in the current clamp mode with perforated patch techniques (EPC-10, HEKA Elektronik, Lambrecht, IN, USA). The borosilicate glass electrodes had tip resistances between 3 and 5 Ω. For I_Na_ recording, the bath solution contained the following (in mM): NaCl 140, CsCl 5.4, CaCl_2_ 1.8, MgCl_2_ 2, nifedipine 0.002 and HEPES 5 (pH 7.3 with NaOH). The pipette solution contained the following (in mM): NaCl 5, CsCl 133, MgATP 2, tetraethylammonium-chloride 20, EGTA 10 and HEPES 5 (pH 7.3 with CsOH). The current was elicited 5 min. after cell rupture by 300 msec. pulses ranging from −70 mV to +40 mV with an increment of 10 mV from a holding potential of −120 mV. For I_K1_ recording, the bath solution contained the following (in mM): NaCl 136, KCl 5.4, CaCl_2_ 1.8, MgCl_2_ 1, glucose 10, CdCl_2_ 0.2 and HEPES 10 (pH 7.4 with NaOH). The pipette solution contained the following (in mM): KCl 20, KOH 110, asparate 110, MgCl_2_ 1, Na2-ATP 5, EGTA 10 and HEPES 10 (pH 7.25 with KOH). The current was elicited by the 300 msec. pulses, which ranged from −120 mV to +10 mV with an increment of 10 mV from a holding potential of −40 mV, and Ba^2+^ was utilized to differentiate I_K1_ from other K^+^ currents. All experiments were conducted at room temperature. The series resistance and capacitance were compensated, and leak currents were subtracted. The individual currents were normalized to the membrane capacity to control for differences in cell sizes; these currents were expressed as the current density pA/pF. The steady-state activation and data were fit using Boltzmann functions.

### Cell surface biotinylation and Western blotting

The cultured NRVMs were washed with cold PBS and biotinylated with Sulfo-NHS-LC-Biotin (Pierce, Rockford, IL, USA) as previously described 13. The protein concentration was determined using the BCA Protein Assay Kit (Beyotime Institute of Biotechnology, Jiangsu, China). Membrane protein samples were denatured with LDS sample buffer (Invitrogen), fractionated on SDS-PAGE (Invitrogen) and then transferred electrophoretically to polyvinylidene fluoride membranes in 25 mmol/l Tris base, 192 mmol/l glycine and 20% ethanol (0.3 A for 1 hr). The membranes were blocked in Tris-buffered saline with 0.1% Tween 20 and 5% nonfat dry milk and were then incubated with primary antibodies [anti-DNM2, 1:500 (Santa Cruz, Dallas, TX, USA); anti-KCNJ2 1:500 (Santa Cruz); and anti-Nav1.5 1: 500 (GST)] overnight at 4°C. After washing and re-blocking, the membranes were incubated with 1:10,000 fluorogenic secondary antibody (KPL Dylight) and were then visualized with an Odyssey system (Licor, Lincoln, NE, USA). Western blot bands were quantified using the quantity one 4.6 software.

### Statistical analysis

The data are expressed as the mean ± SEM. Statistical significance was determined by one-way anova or the unpaired Student's *t*-test when appropriate. A *P* < 0.05 was considered statistically significant.

## Results

### Acute ischaemic insults reduce the expression of endogenous DNM2 in cardiomyocytes

**Figure 1:**
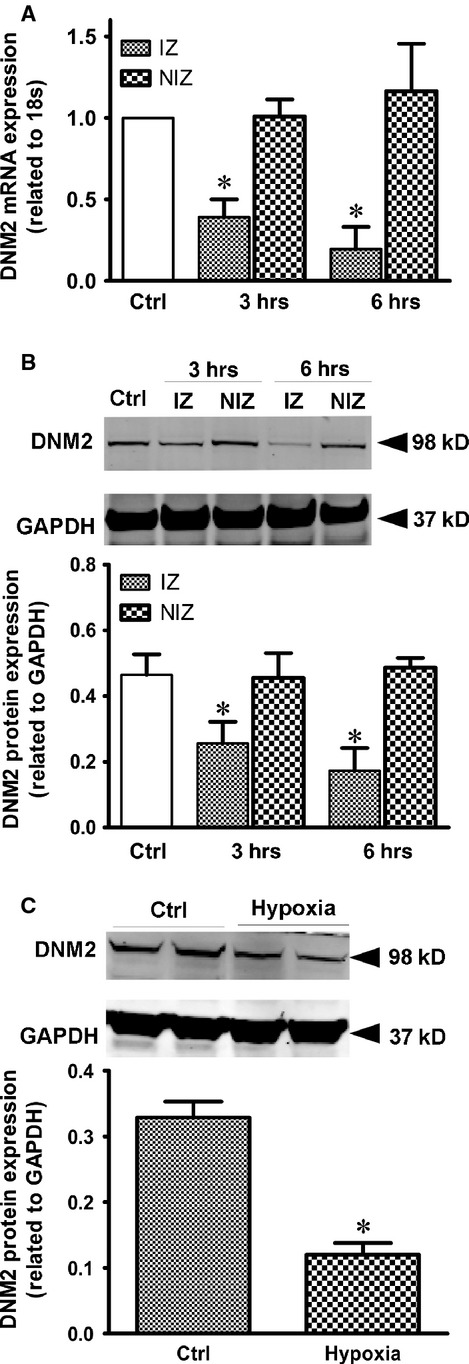
Endogeneous DNM2 is down-regulated in cardiomyocytes subjected to ischaemic stress. (A) Ischaemia induced the decrease in the mRNA expression of DNM2 in rat cardiac tissues. Ctrl: Control, IZ: ischaemic zone, NIZ: non-ischaemic zone. *P < 0.05 compared with the related NIZ. (B) Effects of acute ischaemia on the protein expression of DNM2 in rat cardiac tissues. *Top*, typical Western blots; *bottom*, pooled data (*n* = 3 per group). *P < 0.05 compared with the related NIZ. (C) Hypoxia induced the reduction in DNM2 protein of rat cardiomyocytes. *Top*, typical Western blots; *bottom*, pooled data (*n* = 4 per group). *P < 0.05 compared with ctrl.

To investigate the role of DNM2 in ischaemic cardiac arrhythmias, we first established a rat model of acute myocardial infarction *via* left anterior descending artery ligation. In this model, there are two distinctly active arrhythmogenic periods; one is from immediately after ligation (time 0) to 30 min. following ligation, and the other extends from 90 min. to 9 hrs after ligation 14,20,21. Neural regulation plays a major role in the initiation of arrhythmias in the first of these periods. During the second period, intracellular chemical and electrophysiological imbalances become more important. We recorded ECGs 4–5 hrs after ligation and harvested ischaemic tissue samples at two time-points (3 and 6 hrs post-MI) for further quantitative PCR and Western blot experiments. The mRNA and protein expression levels of DNM2 were markedly reduced in the ischaemic region compared with the non-ischaemic region (Fig.1A and B). However, the protein expression of DNM2 did not change significantly in non-ischaemic tissues.

Given that DNM2 is ubiquitously expressed in various cells, the reduction in DNM2 in cardiac tissue may occur in non-cardiomyocytes. To preclude this possibility, we isolated cardiomyocytes and analysed the cardiomyocyte DNM2 expression levels under ischaemic conditions. As shown in Figure1C, the DNM2 protein expression significantly decreased in cardiomyocytes that were subjected to 12 hrs of hypoxia with 1% O_2_. These results suggest that DNM2 is sensitive to and down-regulated by the ischaemic insults.

### DNM2 defect contributes to ventricular arrhythmias

**Figure 2:**
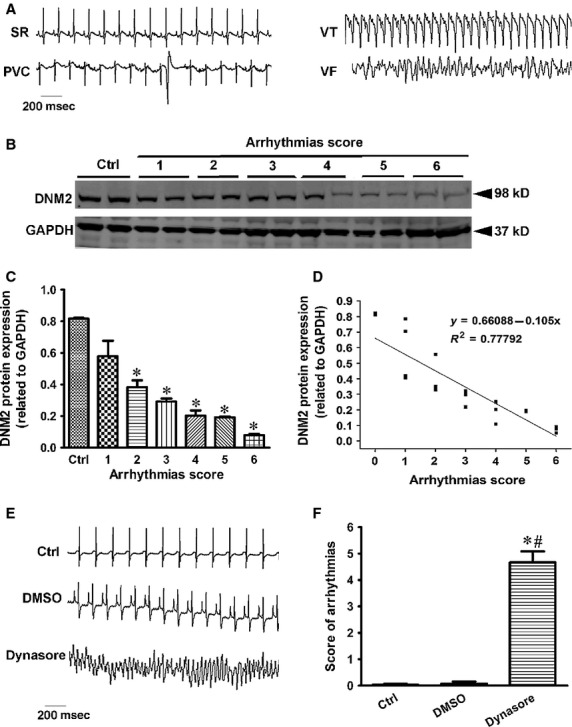
DNM2 inactivation triggers cardiac arrhythmogenesis. (**A**) ECG examples of spontaneous arrhythmias in the rats with acute myocardial infarction, for example, sinus rhythm (SR),ventricular tachycardia (VT) and ventricular fibrillation (VF). (**B**–**C**) Western blots of DNM2 in ischaemic ventricular tissues of rat with different arrhythmias score. (**B**) examples of Western blot bands; (**C**) pooled data from B (*n* = 4 per group). *P < 0.05 *versus* ctrl. (**D**) Linear regression analysis for DNM2 protein expression levels and arrhythmias severity. (*n* = 27). (**E**) Typical ECG recording for isolated intact rat hearts with or without dynasore (15 μM) for 10 min., severe arrhythmias occurred in the dynasore-treated hearts but not in the vehicle DMSO group. (**F**) Arrhythmias scoring analysis in three groups (*n* = 6 per group). *P < 0.05 *versus* ctrl; ^#^*P* < 0.05 *versus*DMSO.

To determine the role of DNM2 in the occurrence and development of ischaemic arrhythmias, we further analysed DNM2 protein expression in the samples with different arrhythmia scores. We found that DNM2 protein expression was reduced in the ischaemic ventricular tissues and the reduction was in linearly correlated with the severity of the arrhythmia (Fig.2A–D). In another set of experiments, we used a specific inhibitor of DNM to verify the direct association of DNM2 reduction with arrhythmogenesis. Using Langendorff perfusion of isolated intact rat hearts with or without dynasore (15 μM), we found that the inhibition of DNM2 triggered arrhythmias, whereas arrhythmias were seldom observed in the hearts that were perfused with the vehicle DMSO (Fig.2E and F). Conclusively, DNM2 reductions could cause severe arrhythmias.

### DNM2 down-regulation increases the action potential amplitude and prolongs the re-polarization duration in ventricular myocytes

**Figure 3:**
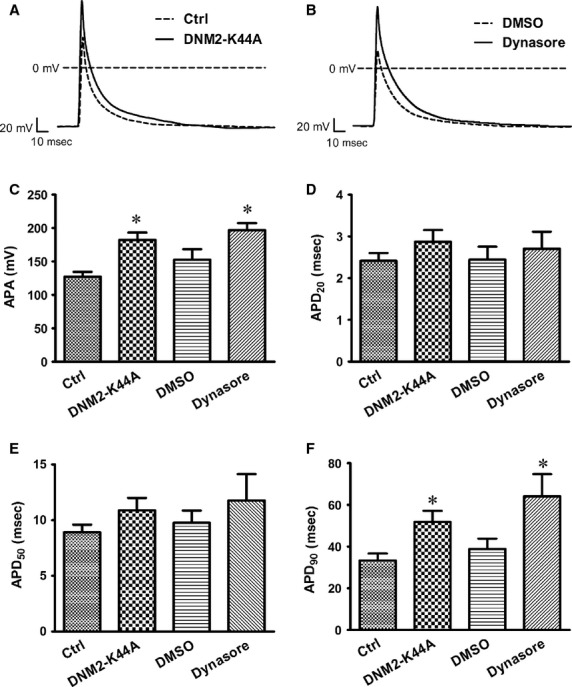
DNM2 deficiency changes AP in ventricular myocytes. (**A**) Representative AP recordings from Ctrl (control vector) and DNM2-K44A overexpression cells. (**B**) Representative AP recordings from DMSO-and dynasore-treated myocytes. (**C**–**F**) Statistic analysis for action potential amplitude (APA) action potential duration at 20% (APD_20_), APD_50_, APD_90_. *P < 0.05 *versus*NC or DMSO.

Abnormal action potential (AP) morphology underlies cardiac arrhythmogenesis 8,22,23. Using the isolated neonatal rat ventricular cardiomyocytes (NRVMs), we analysed the potential effects of a DNM2 deficiency on APs. We cultured NRVMs for 3 days and then transfected them with a DNM2-K44A adenovirus for 24 hrs. Before the patch-clamp experiments, we re-adhered the cultured cells for three hours and then recorded their APs or other currents. As shown in Figure3, the overexpression of DNM2-K44A, a dominant-negative form of DNM2, significantly increased the AP amplitude (APA; from 127.3 ± 7.175 pA/pF, *n* = 21 to 182.2 ± 11.13 pA/pF, *n* = 29) and prolonged the AP duration at 90% re-polarization level (APD_90_) (from 33.30 ± 3.386 msec., *n* = 20 to 51.86 ± 5.309 msec., *n* = 27). Similar AP changes were also observed in cardiomyocytes that were treated with dynasore (15 μM for 24 hrs). There were no significant changes in the APD_20_ or APD_50_ in both cases treated with DNM2-K44A overexpression and pharmacological inhibition of DNM2. DNM2 defects could both induce the increase in the AP amplitude and prolong AP re-polarization.

### DNM2 defects increase Nav1.5 but decrease Kir2.1 channel currents

**Figure 4:**
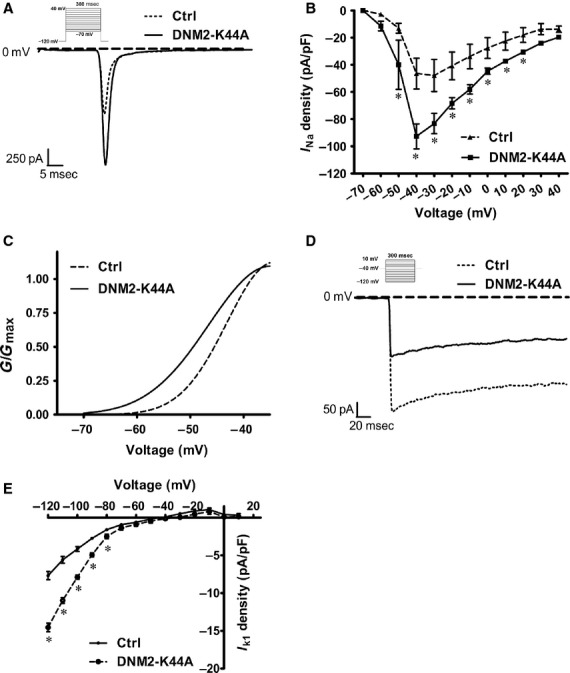
Overexpression of DNM2-K44A increases I_Na_ and decreases I_K1_ in cardio myocytes. (**A**) The I_Na_ current traces under −40 mV from cardiomyocytes expressing DNM2-K44A and control vector. Inset showed the voltage protocols and scale bars. Dashed line indicated zero baseline. (**B**–**C**) The current density of I_Na_ (*n* = 5 per group) (**B**), and activation curves (**C**). (**D**) The I_K1_ current traces under −40 mV from cardiomyocytes expressing DNM2-K44A and control vector. Inset showed the voltage protocols and scale bars. Dashed line indicated zero baseline. (**E**) The current density of I_K1_ (*n* = 9 per group).

Inward sodium current mediates the rapid phase 0 depolarization, and I_K1_ is the most influential in phase 4 23. Therefore, we detected the whole-cell currents of Nav1.5 (SCN5A) and Kir2.1 (KCNJ2) channels in NRVMs. For the Nav1.5 channel, the overexpression of DNM2-K44A increased the sodium current (I_Na_) in NRVMs (Fig.4A). The peak INa density (pA/pF) increased by 44.2% in the DNM2-K44A group compared with the control group (Fig.4B). The INa increase was accompanied by a left shift in the voltage-dependence of steady-state activation, as illustrated in Figure4C.

For the Kir2.1 channel, whole-cell patch-clamp recordings from isolated cardiomyocytes revealed a manifold down-regulation of inward I_K1_ currents. Figure4D and E showed that the inward I_K1_ density measured at −120 mV in cardiomyocytes overexpressing DNM2-K44A was decreased twofold.

### Aberrant membrane translocation of the Nav1.5 and Kir2.1 channel proteins accounts for cardiomyocyte electrophysiological disorders induced by DNM2 reduction

**Figure 5:**
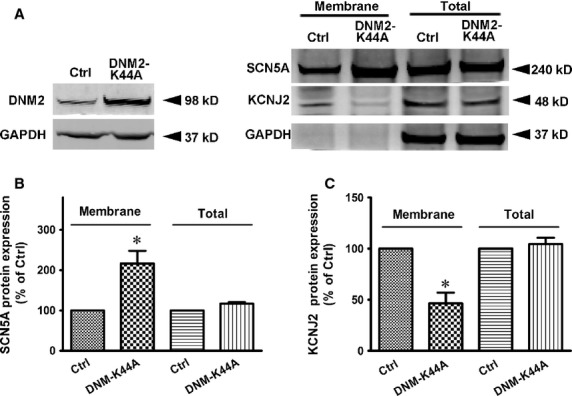
DNM2 regulates the membrane trafficking of Nav1.5 and Kir2.1. (**A**) Effects of DNM2 on membrane protein levels of Nav1.5 and Kir2.1. *Left*, Western blots for DNM2-K44A and control vector. *Right*, typical Western blots showing membrane and total protein levels of Nav1.5 and Kir2.1channels. (**B**–**C**) Pooled data from (A) (*n* = 3 per group). Ctrl: control. *P < 0.05 *versus* Ctrl.

Considering the intrinsic membrane-remodelling features of DNM2, we performed Western blot experiments to detect the protein levels of Nav1.5 and Kir2.1 on the surface of NRVMs. Figure5A through C illustrates that the Nav1.5 membrane protein level was increased and the KCNJ2 membrane protein level was decreased in the DNM2-K44A-overexpressing cardiomyocytes, while the total protein levels of the two channels did not change. This finding suggests that DNM2 influences the membrane trafficking of the Nav1.5 and Kir2.1 channels. Thus, DNM2 influences the function of Nav1.5 and Kir2.1 by regulating their membrane trafficking.

## Discussion

Our present study provides evidence for the role of DNM2 in ischaemic ventricular arrhythmia, which is a common cardiac disease. First, we found that DNM2 is sensitive to compromised metabolic conditions in the heart. Acute myocardial ischaemia induces the reduced expression of myocardial DNM2, which correlates with the incidence of ventricular arrhythmias. Second, we identified that DNM2 plays an important role in regulating the electrophysiological homoeostasis of cardiomyocytes. Reduced DNM2 expression and activity in a single cardiomyocyte results in AP amplitude increases and in a prolonged duration of re-polarization. Correspondingly, enhanced opening of the Nav1.5 channels and depressed I_K1_ currents were observed in DNM2-defective cardiomyocytes. Finally, we uncovered that decreased DNM2 induces the aberrant membrane translocation of the Nav1.5 and Kir2.1 channel proteins.

The association of DNM2 defects with human disease has been previously identified in Charcot–Marie-Tooth peripheral neuropathy 24 and centronuclear myopathy 25–27, both of which involve DNM2 gene loss-of-function mutations. More recently, the DNM2 gene has been described as a susceptibility gene for late onset Alzheimer disease, and the DNM2 mRNA levels are decreased in the brains of these patients 28,29. Our group first reported that DNM2 could mediate heart failure by modulating the Ca^2+^-dependent apoptotic death of cardiomyocytes 12, indicating that DNM2 plays an important role in cardiac disease. Herein, we found that DNM2 expression is sensitive to cardiac metabolic compromise and that there is a rapid reduction in both the mRNA and protein levels of DNM2 in both an ischaemic animal model and oxygen-starved cardiomyocytes. These changes in DNM2 are strongly related to the occurrence of arrhythmias. Furthermore, we found that pharmacological inhibition of DNM2 in isolated, intact rat hearts induces severe arrhythmias *in vitro*. Therefore, our present study establishes a tight link between DNM2 reduction and ischaemic cardiac arrhythmogenesis.

Ischaemic insult leads to a host of complex changes in transsarcolemma ion gradients, ion channel expression and function, and mitochondrial/metabolic properties, all of which combine to form an arrhythmia-promoting environment 29. In this context, abnormal AP morphology and ion channel remodelling are the most important mechanisms in the initial process of ischaemic arrhythmias 30. For instance, a decrease in I_K1_ and the activation of I_Na_ may be responsible for the time-dependent diastolic depolarization. When diastolic depolarization is sufficient to reach a threshold, it will result in sustained arrhythmias 8,31,32. In our present study, DNM2 reduction in cardiomyocytes resulted in increased I_Na_ and decreased I_K1_ along with the corresponding changes in the AP and occurrence of ischaemic ventricular arrhythmias. As for the left-shifted G/Gmax curve of I_Na_, it is possible that DNM2 reduction may also affect the voltage-sensitive opening of Na^+^ channels through an indirect action on the channel, for instance, phosphorylated modification. Meanwhile, these changes in the activation dynamics of Na channel might facilitate the influx of Na ion *via* the channel, and amplify the increase in the APA. Moreover, our previous results suggest that DNM2 reduction can increase the expression of both the Na^+^/H^+^ exchanger-1 and L-type Ca^2+^ channel on the membrane surface, which is consistent with the changes underlying ischaemic arrhythmias 12,13. Collectively, DNM2 and its multi-ion channel properties may be critical for establishing ischaemic cardiac arrhythmias.

Dynamin-2 is characterized by membrane remodelling and by promoting the formation of endocytic vesicles *via* its interactions with pleckstrin homology domains; DNM2 can also guide lipid remodelling through hemifission intermediates 33,11,34. Abnormal membrane trafficking is an important pathophysiological mechanism for DNM2-related diseases 11,35. Given that DNM2 reduction influenced the membrane protein expression of the Nav1.5 and Kir2.1 channels but did not affect the total protein expression, DNM2 seems to regulate the function of these channels through their trafficking. Interestingly, a reduction in DNM2 had the opposite effect on Nav1.5 *versus* Kir2.1. A DNM2 deficiency may limit the retrograde trafficking of the Nav1.5 channel, elevating its membrane expression through inhibiting the formation of endocytic vesicles and decreasing the Nav1.5 channel recycling. However, we cannot explain the DNM2-mediated down-regulation of the Kir2.1 channel membrane expression. Future studies should be performed to elucidate the molecular mechanisms for the differential regulation of the inward and inward rectified channels.

Ischaemic cardiac arrhythmias have always been and continue to be a major public health problem for which the available drugs are not completely effective; instead, the treatments are often pro-arrhythmic. A potential reason is that treatment with medication targeting a single ion channel usually induces an electric imbalance. The unique, multi-ion channel, targeting feature of DNM2 in the myocardium may offer a more effective approach for treating acute ischaemic arrhythmias.

In summary, the membrane-remodelling effect of DNM2 on multiple ion channels underlies its vital function in cardiac electrophysiology. Reductions in DNM2 with an ischaemic insult may alter the membrane expression and opening of ion channels, leading to acute ischaemic arrhythmias.
